# Plasma Neutrophil Gelatinase-Associated Lipocalin as a Predictor of Cardiovascular Events in Patients with Chronic Kidney Disease

**DOI:** 10.1155/2016/8761475

**Published:** 2016-03-14

**Authors:** Midori Hasegawa, Junichi Ishii, Fumihiko Kitagawa, Hiroshi Takahashi, Kazuhiro Sugiyama, Masashi Tada, Kyoko Kanayama, Kazuo Takahashi, Hiroki Hayashi, Shigehisa Koide, Shigeru Nakai, Yukio Ozaki, Yukio Yuzawa

**Affiliations:** ^1^Department of Nephrology, Fujita Health University School of Medicine, 1-98 Dengakugakubo, Kutsukakecho, Toyoakeshi, Aichi 470-1192, Japan; ^2^Laboratory of Clinical Medicine, Department of Joint Research, Fujita Health University School of Medicine, 1-98 Dengakugakubo, Kutsukakecho, Toyoakeshi, Aichi 470-1192, Japan; ^3^Laboratory of Clinical Medicine, Fujita Health University, 1-98 Dengakugakubo, Kutsukakecho, Toyoakeshi, Aichi 470-1192, Japan; ^4^Division of Medical Statistics, Fujita Health University Hospital, 1-98 Dengakugakubo, Kutsukakecho, Toyoakeshi, Aichi 470-1192, Japan; ^5^Department of Cardiology, Fujita Health University School of Medicine, 1-98 Dengakugakubo, Kutsukakecho, Toyoakeshi, Aichi 470-1192, Japan

## Abstract

*Background*. Our aim was to assess plasma neutrophil gelatinase-associated lipocalin (NGAL) as a predictor of cardiovascular (CV) events in patients with chronic kidney disease (CKD) and no history of CV events.* Methods*. This was a prospective observational cohort study of 252 patients with predialysis CKD. CV events were defined as CV death, acute coronary syndrome, and hospitalization for worsening heart failure, stroke, and aortic dissection.* Results*. During a median follow-up period of 63 months, 36 CV events occurred. On Cox stepwise multivariate analysis, plasma NGAL and B-type natriuretic peptide (BNP) were significant predictors of CV events. Kaplan-Meier incidence rates of CV event-free survival at 5 years were 96.6%, 92.9%, 85.9%, and 61.3%, respectively, among quartiles of plasma NGAL (*P* < 0.0001). The C-index for the receiver-operating characteristic curves for CV events was greater when plasma NGAL was added to an established risk model (0.801, 95% CI 0.717–0.885), compared to the model without plasma NGAL (0.746, 95% CI 0.653–0.840, *P* = 0.021).* Conclusion*. Elevated plasma NGAL could predict future CV events in CKD patients with no history of CV events and add incremental value to the established risk model.

## 1. Introduction

Individuals with chronic kidney disease (CKD) are at extremely high cardiovascular (CV) risk [1–4]. CKD contributes to decreased cardiac function, ventricular hypertrophy, and diastolic dysfunction [5]. Biomarkers associated with adverse CV events are useful to detect high risk patients [6–8]. Human neutrophil gelatinase-associated lipocalin (NGAL) was initially identified as a protein isolated from the secondary granules of human neutrophils [9]. In 2003, Mishra et al. reported that NGAL could be detected in the first urine output after ischemia, in both mouse and rat models of acute renal failure [10]. In 2005, Mori et al. clarified that NGAL accumulates in the human kidney cortical tubules and in the blood and urine after nephrotoxic and ischemic injury [11], and Mishra et al. reported that the NGAL concentration in urine and serum represents a sensitive, specific, and highly predictive early biomarker for acute renal injury after cardiac surgery [12]. In the ensuing years, many studies have reported the utility of NGAL as a biomarker of acute kidney injury (AKI) [13].

Recent studies have reported that NGAL may be a biomarker for heart failure as well as renal failure [14, 15]. However, research has not addressed the clinical significance of plasma NGAL as a biomarker of CV risk in patients with CKD who are not on dialysis. The purpose of this study is to evaluate plasma NGAL as a predictor of CV events in patients with CKD.

## 2. Patients and Methods

### 2.1. Study Participants

This prospective cohort study was approved by the Ethics Committee of the Fujita Health University School of Medicine. Patients were enrolled between February 2009 and September 2010. Subjects were eligible for inclusion if they met the following criteria: (1) diagnosis of CKD, defined as having an estimated glomerular filtration rate (eGFR) lower than 60 mL/min/1.73 m^2^; (2) not receiving dialysis; and (3) no history of CV events. CV events regarding past medical history and outcome assessment were defined as acute coronary syndrome, hospitalization for worsening heart failure, stroke, and dissection of aorta, and CV death. Patients were excluded if they presented with fever or signs of infectious diseases, for less than one year. The eGFR was calculated using the Modification of Diet in Renal Disease Study formula for the Japanese population [16].

### 2.2. Measurement of Biomarkers

Blood samples collected for measurement of plasma NGAL were centrifuged at 1000 ×g at 4°C for 15 minutes and stored at −80°C until assayed. Urine samples were also stored at −80°C until assayed. NGAL was measured using enzyme-linked immunosorbent assays (BioPorto Diagnostics, Gentofte, Denmark).

### 2.3. Outcomes Assessment

All patients were observed clinically from enrollment until conclusion of follow-up in December 2014. CV events were assessed by physicians who were blinded to patients' plasma and urinary NGAL levels. Patients were identified as having diabetes if their medical records contained documentation of previous history of diabetes, diagnosis of diabetes on admission, or use of an oral antihyperglycemic agent or insulin at the time of hospital admission.

### 2.4. Statistical Analyses

Statistical analyses were conducted using the Statistical Package for the Social Sciences, Version 19.0 for Windows (SPSS, Chicago, IL, USA). Data are presented as medians and interquartile ranges (IQR). The relationship between the plasma NGAL and other baseline valuables was studied by linear regression analysis. Multivariate regression model including variables with *P* < 0.05 in the individual model was used to determine which covariates were independently associated with log plasma NGAL levels. Differences in event-free survival by quartile of plasma NGAL were examined using the Kaplan-Meier method and compared using a log-rank test. Hazard ratios (HR) and 95% confidence intervals (CI) were calculated for each factor with Cox proportional hazards analysis. To identify independent predictors of CV events, all baseline variables with *P* < 0.05 in the univariate analysis were entered into a multivariate model. In addition, to assess if CV events could be predicted more accurately after the addition of plasma NGAL to the model, the C-index for the receiver-operating characteristic (ROC) curves was calculated using a logistic multivariate model. Differences were considered statistically significant at *P* < 0.05.

## 3. Results

### 3.1. Study Population

The study enrolled 252 patients. Characteristics of the study population are summarized in Table 1. The median age of patients was 67 (60–75) years, and 59.1% were male. The distribution of patients among the CKD grades was 25.4% of G3a, 14.7% of G3b, 35.7% of G4, and 24.2% of G5.

### 3.2. Relationships between Urinary and Plasma Neutrophil Gelatinase-Associated Lipocalin

**Figure 1:**
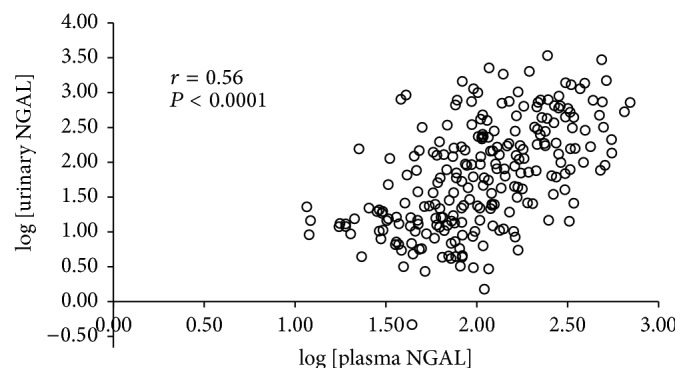
Plot of log plasma neutrophil gelatinase-associated lipocalin (NGAL) and log urinary NGAL. There was a significant correlation between plasma and urinary NGAL (*r* = 0.56, *P* < 0.0001).

The scatter plot of log (plasma NGAL) and log (urinary NGAL) is shown in Figure 1. The correlation coefficient was 0.56, *P* < 0.0001.

### 3.3. Correlates of Plasma Neutrophil Gelatinase-Associated Lipocalin Levels

Correlation between clinical and laboratory variables and plasma NGAL levels are shown in Table 2. The strongest individual correlations were observed with eGFR. Moderate associations were found with hemoglobin (Hb), urinary NGAL, and the urinary albumin creatinine ratio (UACR). Weak associations were found with B-type natriuretic peptide (BNP) and high-density lipoprotein- (HDL-) cholesterol. Four variables were independently associated with NGAL levels in the multivariate analysis: eGFR, HDL-cholesterol, UACR, and BNP.

### 3.4. Prognostic Value of Plasma Neutrophil Gelatinase-Associated Lipocalin for Cardiovascular Events

**Figure 2:**
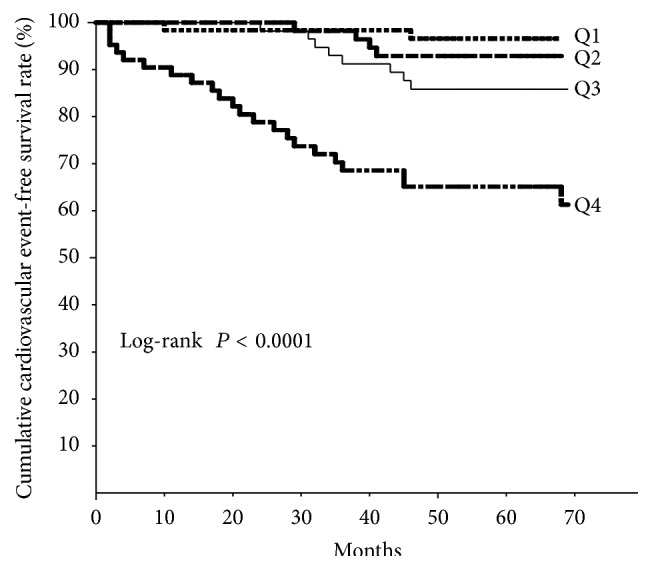
Kaplan-Meier curves for cardiovascular event-free survival by quartile (Q1–Q4) of plasma neutrophil gelatinase-associated lipocalin level. A clear difference among quartiles is evident. Quartile 1 (Q1): ≤60 ng/mL, quartile 2 (Q2): 61–104 ng/mL, quartile 3 (Q3): 105–193 ng/mL, and quartile 4 (Q4): ≥194 ng/mL.

During a median follow-up period of 63 months, 36 CV events (14.3%) occurred. Twenty-two consisted of admission due to heart failure, 5 were acute myocardial infarction, 5 were cerebral infarction, 3 were aortic dissection, and 1 was unstable angina pectoris.  Table 3 shows Cox analysis for CV events. On univariate analysis, age, plasma NGAL, BNP, eGFR, hemoglobin, high-sensitivity C-reactive protein (hsCRP), and UACR were significant predictors of CV events. When we performed Cox stepwise multivariate analysis including all variables with *P* < 0.05 on univariate analysis, plasma NGAL and BNP were significant predictors of CV events. Five-year CV event-free survival rates in quartile 1 (Q1; ≤60 ng/mL), quartile 2 (Q2; 61–104 ng/mL), quartile 3 (Q3; 105–193 ng/mL), and quartile 4 (Q4; ≥194 ng/mL), calculated by the Kaplan-Meier method, were 96.6%, 92.9%, 85.9%, and 61.3%, respectively (*P* < 0.0001, Figure 2).

### 3.5. Receiver-Operating Characteristic Curves for Cardiovascular Events

**Figure 3:**
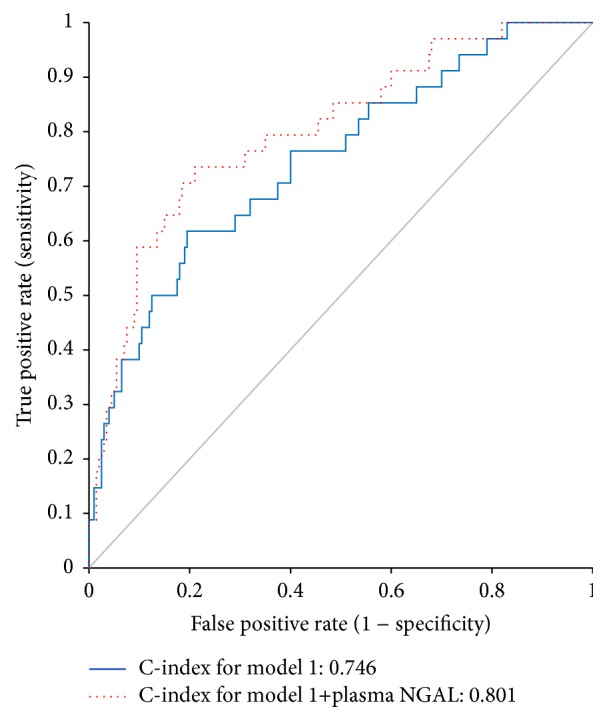
Receiver-operating characteristic curve for cardiovascular events in model 1 and model 2. Model 1: BNP + hsCRP + UACR + Hb + eGFR + age. Model 2: Model 1 + plasma NGAL. BNP, B-type natriuretic peptide; eGFR, estimated glomerular filtration rate; hsCRP, high-sensitivity C-reactive protein; Hb, hemoglobin; NGAL, neutrophil gelatinase-associated lipocalin; UACR, urinary albumin creatinine ratio.

Figure 3 shows the ROC curves for CV events. The addition of plasma NGAL to the predictive model based on BNP, hsCRP, UACR, Hb, eGFR, and age had an effect on model discrimination as measured by the C-index (0.746 (95% CI 0.653–0.840) to 0.801 (95% CI 0.717–0.885), *P* = 0.021).

## 4. Discussion

Our primary finding was that plasma NGAL is an independent predictor of CV events. Plasma NGAL therefore provides incremental value to an established risk model that includes BNP, in patients with CKD and no previous history of CV events. Neutrophils are the major source of circulating NGAL in both normal and infected states, whereas, during AKI, blood and urinary NGAL derive mainly from the kidney [17]. In an AKI model, NGAL is expressed in the distal nephron. In a study of diabetic mice induced by streptozotocin, which is a model of slowly progressive CKD, urinary NGAL appeared to derive primarily from impaired reabsorption in the proximal tubules [18]. Recent studies indicate that chronic renal damage could influence the physiological balance of NGAL in a manner similar to that observed for acute injury conditions. Chronically damaged tubular cells may produce a large quantity of NGAL because of active chronic stress-induced production by the injured tubular cells [19]. Plasma NGAL measurement has been reported to be influenced by other coexisting factors, including inflammatory conditions [20], anemia, and hypoxia [21]. NGAL expression also occurs in a failing myocardium [22] and is present in atherosclerotic plaque and human abdominal aortic aneurysms [23, 24]. Clinically, divergent results have been observed with regard to ventricular structure and function. Serum NGAL was weakly correlated with left ventricular (LV) ejection fraction in one small study [25], while other studies found no association between plasma or urine NGAL and echocardiographic indices of LV cardiac structure or left or right ventricular systolic function [22, 26]. In another study, plasma NGAL levels were modestly associated with indices of diastolic dysfunction, but not after adjustment for renal function [22]. In our cohort, plasma NGAL had no significant correlation with LV ejection fraction. Plasma NGAL was independently correlated with eGFR, HDL-cholesterol, UACR, and BNP. Thus plasma NGAL may reflect a variety of factors associated with CV events.

In terms of the CV-related prognostic value of NGAL, several conflicting results have been reported in different cohorts [24–27]. Lindberg et al. found that high plasma NGAL independently predicts major adverse cardiac events in ST-segment elevation myocardial infarction patients treated with primary percutaneous coronary intervention [27]. Falke et al. reported that plasma NGAL was a significant independent predictor of long-term CV mortality in patients with acute cerebral ischemia [28]. Daniels et al. reported that plasma NGAL was a significant predictor of mortality and CV events in community-dwelling older adults, independent of traditional risk factors and kidney function, and added incremental value to N-terminal pro-B-type natriuretic peptide (NT-proBNP) and CRP [29]. On the contrary, Nymo et al. reported that NGAL added no significant information to NT-proBNP and GFR in a multivariate model for primary and secondary end-points in chronic heart failure [30]. In our study, we used BNP, rather than NT-proBNP, to detect natriuretic peptide levels. BNP is a neurohormone secreted mainly in the cardiac ventricles in response to volume expansion and pressure overload. Plasma BNP levels have a strong correlation with LV filling pressure and increase in proportion to the severity of LV systolic dysfunction and diastolic dysfunction [31, 32]. Unlike NT-proBNP, plasma BNP levels are relatively independent of GFR. In addition, levels of BNP have been reported to have powerful predictive potential for heart failure in nondialysis CKD patients [33].

Recently, we reported that urinary NGAL was a predictor of CV events in CKD patients, during a median follow-up period of 33 months [34]. That study cohort contained patients who had a history of CV events. In the present study, patients with a history of CV events were excluded, and the median follow-up period was extended to 63 months. There was a significant correlation between plasma NGAL and urinary NGAL (*r* = 0.57, *P* < 0.0001). However, there were also discrepancies in these two parameters for some patients. Whereas urinary NGAL seems to be closely linked to kidney function and kidney damage, circulating NGAL levels may also mirror other systemic conditions that we described above. This might explain why urinary NGAL did not remain as a significant predictor of CV events when adjusting for confounders, including eGFR.

## 5. Limitations

This study has several limitations. First, we used a single-center design. Larger multicenter studies might confirm our findings. Second, we could not evaluate any association between NGAL and cardiac death or all-cause mortality because of the small number of deaths. Third, we could not perform separate analysis of each CV end-point, because when separated, each event was too infrequent to be included in multivariate analysis.

## 6. Conclusions

In conclusion, plasma NGAL is an important independent predictor of CV events in patients with CKD and no previous CV events. Measuring plasma NGAL could contribute clinically significant information to the evaluation of patients at high risk of CV events.

## Figures and Tables

**Table 1:** Clinical characteristicof 252 CKD patients.

	All patients (*n* = 252)
Male (%)	59.1
Age (years)	67 (60–75)
Diabetes (%)	35.7
Smoking (%)	37.9
Systolic BP (mmHg)	130 (122–142)
BMI (kg/m^2^)	22.3 (20.2–24.7)
CKD grade (%)	
G3a	25.4
G3b	14.7
G4	35.7
G5	24.2
Plasma NGAL (ng/mL)	105 (60–194)
Urinary NGAL (*μ*g/gCr)	65 (15–235)
BNP (pg/mL)	30 (17–71)
eGFR (mL/min/1.73 m^2^)	25.1 (15.2–38.5)
Hemoglobin (g/dL)	11.3 (9.8–12.7)
hsCRP (mg/L)	0.6 (0.2–1.5)
UACR (mg/gCr)	338 (102–1321)
TG (mg/dL)	137 (103–193)
LDL-C (mg/dL)	109 (87–129)
HDL-C (mg/dL)	54 (44–68)
ECG^a^	
LVH (%)	6.1
ST-T change (%)	9.8
EF (%) by UCG^b^	60.0 (57.0–64.0)
ARB or ACEi (%)	71.0
Statins (%)	29.8

^a^ *n* = 194; ^b^ *n* = 151.

ACEi, angiotensin-converting enzyme inhibitor; ARB, angiotensin receptor blocker; BP, blood pressure; BMI, body mass index; BNP, B-type natriuretic peptide; ECG, electrocardiogram; CKD, chronic kidney disease; EF, ejection fraction; eGFR, estimated glomerular filtration rate; HDL-C, high-density lipoprotein-cholesterol; hsCRP, high-sensitivity C-reactive protein; LDL-C, low-density lipoprotein-cholesterol; LVH, left ventricular hypertrophy; NGAL, neutrophil gelatinase-associated lipocalin; TG, triglyceride; UACR, urinary albumin creatinine ratio; UCG, ultracardiography.

**Table 2:** Individual and multivariable covariates of log plasma NGAL levels.

Variable	Individual		Multivariate^*∗*^	
	*r*	*P*	*β*	*P*
Age	0.063	0.32		
Systolic BP	0.14	0.024		
BMI	0.043	0.52		
log urinary NGAL	0.56	<0.0001		
logBNP	0.34	<0.0001	0.12	0.010
logeGFR	−0.73	<0.0001	−0.59	<0.0001
logHb	−0.50	<0.0001		
loghsCRP	0.064	0.31		
logUACR	0.45	<0.0001	0.17	0.001
logTG	−0.090	0.16		
logLDL-C	−0.13	0.049		
logHDL-C	−0.23	<0.0001	−0.20	<0.0001
EF (%) by UCG^b^	−0.14	0.082		

^b^ *n* = 151.

^*∗*^ *R* ^2^ = 0.58; *β* = standardized regression coefficient.

Multivariate model includes all variables with *P* < 0.05 by individual model.

BP, blood pressure; BMI, body mass index; BNP, B-type natriuretic peptide; EF, ejection fraction; eGFR, estimated glomerular filtration rate; HDL-C, high-density lipoprotein-cholesterol; hsCRP, high-sensitivity C-reactive protein; LDL-C, low-density lipoprotein-cholesterol; NGAL, neutrophil gelatinase-associated lipocalin; TG, triglyceride; UACR, urinary albumin creatinine ratio; UCG, ultracardiography.

**Table 3:** Predictors of cardiovascular events.

Variable	Nonadjusted		Adjusted	
	HR (95% CI)	*P* value	HR (95% CI)	*P* value
Male (yes)	0.99 (0.51–1.95)	1.00		
Age (yes)	1.04 (1.01–1.07)	0.01	1.02 (0.99–1.05)	0.24
Diabetes (yes)	1.87 (0.97–3.60)	0.06		
Smoking (yes)	0.84 (0.42–1.65)	0.61		
Systolic BP (mmHg)	1.02 (0.99–1.04)	0.12		
BMI (kg/m^2^)	0.95 (0.86–1.05)	0.35		
Plasma NGAL (ng/mL)	1.005 (1.003–1.007)	<0.0001	1.004 (1.002–1.006)	<0.0001
Urinary NGAL (*μ*g/gCr)	1.00 (0.999–1.001)	0.71		
BNP (pg/mL)	1.005 (1.003–1.006)	<0.0001	1.005 (1.003–1.007)	<0.0001
eGFR (mL/min/1.73 m^2^)	0.96 (0.93–0.99)	0.002	0.99 (0.96–1.04)	0.87
Hemoglobin (g/dL)	0.72 (0.60–0.85)	<0.0001	0.94 (0.78–1.14)	0.54
hsCRP (mg/L)	1.78 (1.32–2.35)	<0.0001	1.33 (0.98–1.82)	0.07
UACR (g/gCr)	1.26 (1.09–1.44)	0.001	1.16 (0.97–1.39)	0.09
Triglyceride (mg/dL)	0.99 (0.99–1.00)	0.36		
LDL-C (mg/dL)	1.00 (0.99–1.01)	0.40		
HDL-C (mg/dL)	0.99 (0.97–1.00)	0.21		
LVH of ECG^a^	1.75 (0.53–5.75)	0.36		
ST-T change of ECG^a^	1.14 (0.35–3.76)	0.83		
EF of UCG^b^	0.94 (0.88–1.00)	0.056		
ARB or ACEi (yes)	0.66 (0.33–1.30)	0.23		
Statins (yes)	0.96 (0.47–1.95)	0.91		

^a^ *n* = 194; ^b^ *n* = 151.

Multivariate model includes all variables with *P* < 0.05 by univariate analysis.

ACEi, angiotensin-converting enzyme inhibitor; ARB, angiotensin receptor blocker; BMI, body mass index; BNP, B-type natriuretic peptide; BP, blood pressure; ECG, electrocardiogram; EF, ejection fraction; eGFR, estimated glomerular filtration rate; HDL-C, high-density lipoprotein-cholesterol; HR, hazard ratio; hsCRP, high-sensitivity C-reactive protein; LDL-C, low-density lipoprotein-cholesterol; LVH, left ventricular hypertrophy; NGAL, neutrophil gelatinase-associated lipocalin; UACR, urinary albumin creatinine ratio; UCG, ultracardiography.
